# Supplementary material for: Impact and Effectiveness of Group Strategies for Supporting Breastfeeding after Birth: A Systematic Review
Source: Int J Environ Res Public Health. 2021 Mar 4;18(5):2550. doi: 10.3390/ijerph18052550 (PMC7967547; doi:10.3390/ijerph18052550)
Supplement: Supplementary file 1 [file ijerph-18-02550-s001.pdf]

International Journal of
Environmental Research and Public Health

MDPI

Supplementary Material

Table S1. PRISMA 2009 Checklist: Recommended items in a systematic review.

Section and topic	Item No.	Checklist item	Reported on page
**TITLE**			
Title:	1	Identify the report as a systematic review, meta-analysis, or both.	1
**ABSTRACT**			
Structured summary	2	Provide a structured summary including, as applicable: background; objectives; data sources; study eligibility criteria, participants, and interventions; study appraisal and synthesis methods; results; limitations; conclusions and implications of key findings; systematic review registration number.	1
**INTRODUCTION**			
Rationale	3	Describe the rationale for the review in the context of what is already known	1–2
Objectives	4	Provide an explicit statement of questions being addressed with reference to participants, interventions, comparisons, outcomes, and study design (PICOS).	2–3
**METHODS**			
Protocol and registration	5	Indicate if a review protocol exists, if and where it can be accessed (e.g., Web address), and, if available, provide registration information including registration number.	-
Eligibility criteria	6	Specify study characteristics (e.g., PICOS, length of follow-up) and report characteristics (e.g., years considered, language, publication status) used as criteria for eligibility, giving rationale.	2–3
Information sources	7	Describe all information sources (e.g., databases with dates of coverage, contact with study authors to identify additional studies) in the search and date last searched.	2–3
Search	8	Present full electronic search strategy for at least one database, including any limits used, such that it could be repeated.	3
Study selection	9	State the process for selecting studies (i.e., screening, eligibility, included in systematic review, and, if applicable, included in the meta-analysis).	4–5
Data collection process	10	Describe method of data extraction from reports (e.g., piloted forms, independently, in duplicate) and any processes for obtaining and confirming data from investigators.	4–5
Data items	11	List and define all variables for which data were sought (e.g., PICOS, funding sources) and any assumptions and simplifications made.	
Risk of bias in individual studies	12	Specify any assessment of risk of bias that may affect the cumulative evidence (e.g., publication bias, selective reporting within studies).	6–7
Summary measures	13	State the principal summary measures (e.g., risk ratio, difference in means).	-
Synthesis or results	14	Describe the methods of handling data and combining results of studies, if done, including measures of consistency (e.g., I^2) for each meta-analysis.	-
Risk of bias across studies	15	Specify any assessment of risk of bias that may affect the cumulative evidence (e.g., publication bias, selective reporting within studies).	6–7

Additional analyses	16	Describe methods of additional analyses (e.g., sensitivity or subgroup analyses, meta-regression), if done, indicating which were pre-specified.	-
**RESULTS**			
Study selection	17	Give numbers of studies screened, assessed for eligibility, and included in the review, with reasons for exclusions at each stage, ideally with a flow diagram.	4–5
Study characteristics	18	For each study, present characteristics for which data were extracted (e.g., study size, PICOS, follow-up period) and provide the citations.	7–13
Risk of bias within studies	19	Present data on risk of bias of each study and, if available, any outcome level assessment (see item 12).	6–7;7–12 Supplementary material
Results of individual studies	20	For all outcomes considered (benefits or harms), present, for each study: (a) simple summary data for each intervention group (b) effect estimates and confidence intervals, ideally with a forest plot.	7–12
Synthesis of results	21	Present results of each meta-analysis done, including confidence intervals and measures of consistency.	-
Risk of bias across studies	22	Present results of any assessment of risk of bias across studies (see Item 15).	-
Additional analysis	23	Give results of additional analyses, if done (e.g., sensitivity or subgroup analyses, meta-regression [see Item 16]).	-
**DISCUSSION**			
Summary of evidence	24	Summarize the main findings including the strength of evidence for each main outcome; consider their relevance to key groups (e.g., healthcare providers, users, and policy makers).	19
Limitations	25	Discuss limitations at study and outcome level (e.g., risk of bias), and at review-level (e.g., incomplete retrieval of identified research, reporting bias).	20
Conclusions	26	Provide a general interpretation of the results in the context of other evidence, and implications for future research.	20
**FUNDING**			
Funding	27	Describe sources of funding for the systematic review and other support (e.g., supply of data); role of funders for the systematic review.	21

From: Moher, D.; Liberati, A.; Tetzlaff, J.; Altman, D.G. The PRISMA Group. Preferred Reporting Items for Systematic Reviews and Meta-Analyses: The PRISMA Statement.PLoS Med. **2009**, *6*(7): e1000097. doi:10.1371/journal.pmed1000097.

Table S2. Methodological quality evaluation of observational studies—STROBE scale.

Section/Topic	Item No.	Rayfield et al. [36]	Undlien et al. [37]	Ruducha et al. [38]	Robinson et al. [39]	Jenkins et al. [40]
**Title and abstract**	1a	✓	x	x	✓	✓
	1b	✓	✓	✓	✓	✓
**Introduction**						
Background/Rationale	2	✓	✓	✓	✓	✓
Objectives	3	✓	✓	✓	✓	✓
**Methods**						
Study design	4	✓	✓	✓	✓	x
Setting	5	✓	✓	✓	✓	✓
Participants	6	-	✓	✓	✓	✓
	6b	✓	-	-	-	-
Variables	7	✓	x	x	✓	x
Data sources/Measurement	8	✓	✓	✓	✓	x
Bias	9	x	x	x	x	x
Study size	10	x	x	x	x	x
Quantitative variables	11	✓	✓	✓	✓	x
Statistical methods	12a	✓	x	✓	✓	x
	12b	✓	✓	✓	✓	x
	12c	x	x	x	x	x
	12d	x	x	x	x	x
	12e	✓	✓	✓	x	x
**Results**						
Participants	13a	✓	✓	x	✓	✓
	13b	-	✓	x	x	x
	13c	x	x	x	x	x
Descriptive data	14a	✓	✓	✓	✓	✓
	14b	x	x	x	x	x
	14c	-	-	-	-	-
Outcome data	15	✓	✓	✓	✓	✓
Main Results	16a	✓	✓	✓	✓	✓
	16b	✓	x	✓	✓	✓
	16c	✓	-	✓	-	-
Other analyses	17	✓	✓	✓	✓	✓
**Discussion**						
Key results	18	✓	✓	✓	✓	✓
Limitations	19	✓	✓	✓	✓	✓
Interpretation	20	✓	✓	✓	✓	✓

Generalizability	21	✓	✓	✓	✓	✓
**Other information**						
Funding	25	✓	x	✓	✓	✓

X: Unidentified item
✓: Identified item
-: Not applicable
From: von Elm, E.; Altman, D.G.; Egger, M.; Pocock, S.J.; Gøtzsche, P.C.; Vandenbroucke, J.P..STROBE Initiative. The Strengthening the Reporting of Observational Studies in Epidemiology (STROBE) statement: guidelines for reporting observational studies. PLoS Med. **2007**,*16*:e296. doi: 10.1371/journal.pmed.0040296.

Table S3. Evaluation of the methodological quality of experimental studies—CONSORT 2010 checklist.

Section/Topic	Item No.	Moudy et al. [28]	Niela-Vilén et al. [29]	Scott et al. [33]	Schreck et al. [34]	Saggurti et al. [30]	Lee et al. [35]	Hazra et al. [32]	M'Liria et al. [31]
Title and abstract	1a	-	✓	-	-	-	-	-	✓
	1b	✓	✓	✓	✓	✓	✓	✓	✓
Background and objectives	2a	✓	✓	✓	✓	✓	✓	✓	✓
	2b	✓	✓	✓	✓	✓	✓	✓	✓
Trial design	3a	✓	✓	x	✓	✓	✓	✓	✓
	3b	x	x	x	x	x	x	x	x
Participants	4a	✓	✓	✓	✓	✓	✓	✓	✓
	4b	✓	✓	✓	✓	✓	✓	✓	✓
Interventions	5	✓	✓	✓	✓	✓	✓	✓	✓
Outcomes	6a	✓	✓	✓	✓	✓	✓	✓	✓
	6b	x	x	x	x	x	x	x	x
Sample size	7a	✓	✓	x	x	✓	✓	✓	✓
	7b	✓	✓	x	x	x	x	x	x
Sequence generation	8a	✓	✓	-	-	✓	-	-	✓
	8b	✓	✓	-	-	✓	-	-	✓
Allocation concealment mechanism	9	x	x	-	-	x	-	-	✓
Implementation	10	✓	x	-	x	✓	x	x	✓
Blinding	11a	x	x	x	x	x	x	x	✓
	11b	-	-	-	-	-	-	-	✓
Statistical methods	12a	✓	✓	✓	✓	✓	✓	✓	✓
	12b	✓	x	x	x	x	✓	✓	✓
Participant flow (a diagram is strongly recommended)	13a	✓	✓	-	x	x	✓	x	✓
	13b	✓	✓	-	x	x	✓	x	✓
Recruitment	14a	✓	✓	✓	✓	✓	✓	✓	✓
	14b	✓	✓	✓	✓	✓	✓	✓	✓

Baseline data	15	✓	✓	x	✓	✓	✓	✓	✓
Numbers analysed	16	✓	✓	✓	✓	✓	✓	✓	✓
Outcomes and estimation	17a	✓	✓	✓	✓	✓	✓	✓	✓
	17b	✓	✓	✓	✓	✓	✓	✓	✓
Ancillary analyses	18	✓	✓	✓	✓	✓	✓	✓	✓
Harms	19	x	x	x	x	x	x	x	x
Limitations	20	x	✓	✓	✓	✓	✓	✓	✓
Generalisability	21	✓	✓	✓	✓	✓	✓	✓	✓
Interpretation	22	✓	✓	✓	✓	✓	✓	✓	✓
Registration	23	x	✓	-	x	x	x	x	x
Protocol	24	x	x	x	x	x	x	x	x
Funding	25	x	✓	✓	✓	x	✓	✓	✓

X: Unidentified item
✓: Identified item
-: Not applicable
From: Schulz, K.F.; Altman, D.G.; Moher, D.; for the CONSORT Group. CONSORT 2010 Statement: updated guidelines for reporting parallel group randomized trials. BMJ. **2010**, *23*:c332. doi: 10.1136/bmj.c332
